# Endothelial-dependent relaxation of α-pinene and two metabolites, myrtenol and verbenol, in isolated murine blood vessels

**DOI:** 10.1152/ajpheart.00380.2023

**Published:** 2023-10-27

**Authors:** L. Jin, Z. Xie, P. Lorkiewicz, S. Srivastava, A. Bhatnagar, D. J. Conklin

**Affiliations:** ^1^Christina Lee Brown Envirome Institute, University of Louisville, Louisville, Kentucky, United States; ^2^Division of Environmental Medicine, Department of Medicine, University of Louisville, Louisville, Kentucky, United States

**Keywords:** endothelium, greenness, monoterpenes, nitric oxide, transient receptor potential ankyrin-1

## Abstract

Epidemiological evidence shows that residential proximity to greenspaces is associated with lower risk of all-cause and cardiovascular mortality; however, the mechanism(s) underlying this link remains unclear. Plants emit biogenic volatile organic compounds such as α-pinene that could elicit beneficial cardiovascular effects. To explore the role of α-pinene more directly, we studied the metabolism and the vascular effects of α-pinene. We found that exposure of mice to α-pinene (1 ppm, 6 h) generated two phase I oxidation metabolites, *cis*- and *trans*-verbenol [(1*R*,2*R*,5*R*)-verbenol and (1 *R*,2*S*,5*R*)-verbenol)] and myrtenol [(1*S*,5*R*)-(+)-myrtenol] that were identified in urine by GC-MS. Precontracted naïve murine male and female aorta and superior mesenteric artery (SMA) were relaxed robustly (60% tension reduction) by increasing concentrations of α-pinene, myrtenol, and verbenol to 0.3 mM, whereas 1 mM α-pinene was vasotoxic. The SMA was six times more sensitive than the aorta to α-pinene. Both myrtenol and verbenol were equally potent and efficacious as parent α-pinene in male and female SMA. The sensitive portion of the α-pinene-, myrtenol-, and verbenol-induced relaxations in male SMA was mediated by *1*) endothelium, *2*) eNOS-derived NO, and *3*) guanylyl cyclase (GC) activity. Moreover, α-pinene activated the transient receptor potential ankyrin-1 (TRPA1) channel whereas the metabolites did not. Endothelial-derived NO regulates blood flow, blood pressure, and thrombosis, and it is plausible that inhaled (and ingested) α-pinene (or its metabolites) augments NO release to mediate the cardiovascular benefits of exposure to greenness.

**NEW & NOTEWORTHY** A common plant-derived biogenic volatile organic compound, α-pinene, and two of its metabolites, myrtenol and verbenol, stimulate vasorelaxation in murine superior mesenteric artery. Both α-pinene- and its metabolites induce vasorelaxation by activation of the endothelium, nitric oxide, and guanylyl cyclase. α-Pinene also activates the transient receptor potential ankyrin-1. Positive associations between greenness exposure and human cardiovascular health may be a result of the vascular action of α-pinene and its metabolites, a novel consideration.

## Graphical abstract

**Figure:**
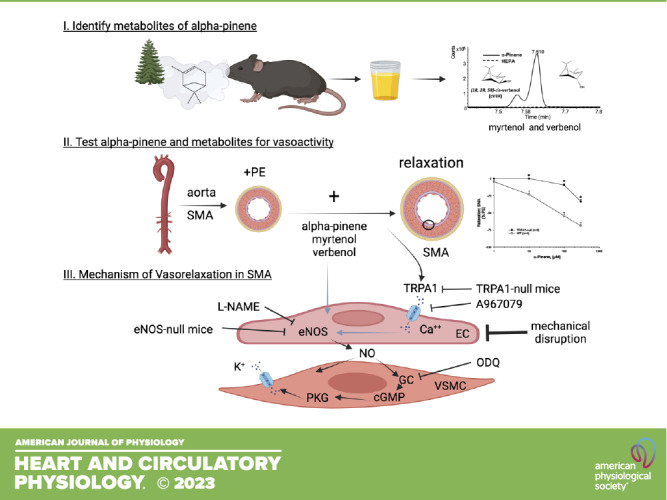

## INTRODUCTION

Vegetation is a primordial feature of the natural environment and human existence is critically dependent on plants and vegetation for oxygen, food, and shelter (1). However, recent urbanization has limited human interactions with vegetation, despite significant resources to maintain gardens, parks, green public spaces, and yard/street trees in our environments. Nonetheless, exposure to greenspace within the natural environment has been linked with improvements in cardiovascular health. In a national study from the United Kingdom, CVD mortality rates were significantly elevated in areas with low levels of greenness compared with areas of higher levels of greenness (2). Similarly, the loss of 100 million ash trees in the northern United States has been reported to be associated with a significant increase in CVD mortality (3), suggesting that greenness may be an important determinant of cardiovascular health. Consistent with this finding, another study of residential greenness and mortality in 108,630 participants shows that those living in areas of the highest levels of greenness had 12% lower mortality rates compared with those in the lowest level of greenness (4). Likewise, a 10-yr longitudinal study of 1.2 million individuals residing in Rome found that greenness is associated with significant reductions in CVD mortality (5). Furthermore, in an 11-yr study of urban individuals in Canada, increased greenness close to one’s residence was found to be significantly associated with reduced CVD mortality and greater benefit against CVD than for other causes of death (6).

Although many environmental and social factors such as a decrease in air, noise, and light pollutions, lower levels of stress, higher physical activity, and greater social cohesion have been posited to account for the salubrious effects of surrounding greenness, the process and mechanisms linking greenness to better health, however, remain unclear. In addition to such environmental and social factors, the health effects of greenness are likely to be mediated by direct inhalation of several vasoactive substances emitted by plants such as the biogenic volatile organic compounds (bVOC) that may directly affect vascular health (7).

Although prior research in rats has shown bVOCs, especially monoterpenes (α-pinene, α-pinene, citronellol, limonene, linalool) reduce blood pressure and heart rate, these studies relied on intravenous, suprapharmacological doses unlikely to be achieved via inhalation (8–12). Whereas inhalation of limonene, linalool, and α-pinene in humans increases high-frequency heart rate variability (HRV) and exerts anti-inflammatory effects (13–15), the role of metabolism (16, 17) or the direct mechanism(s) underpinning these potentially beneficial effects is unclear (18). Monoterpenes are abundant in plants; for example, α-pinene is >42% of a plant (*Seseli pallasii*) extract (19). Moreover, and suggestive of beneficial cardiovascular actions, a *S. pallasii* extract induces vasorelaxation in isolated, precontracted mesenteric artery (19). Because of the abundance of monoterpenes in plant bVOCs, and, specifically, α-pinene with its known relationship with blood pressure, we hypothesized that α-pinene would stimulate relaxation in isolated, precontracted murine blood vessels. In addition, we tested the idea that the metabolites of α-pinene may be vasoactive as well. Study of the mechanisms of vasorelaxation of bVOCs may help us understand how exposure to bVOCs can confer cardiovascular health benefits.

## MATERIALS AND METHODS

### Chemicals

Chemicals were purchased from Sigma-Aldrich and/or other commercial sources as indicated: A-967079 (TRPA1 antagonist; AdooQ; Irvine, CA); acetylcholine chloride (ACh); (1 *R*)-(+)-α-pinene (α-pinene); β-glucuronidase/arylsulfatase; myrtenol; ^13^C-myrtenol (Toronto Research Chemicals, Toronto, Canada); 1 h-[1,2,4]oxadiazolo[4,3-a]quinoxalin-1-one (ODQ); *N*^ω^-nitro-l-arginine methyl ester (l-NAME); l-phenylephrine hydrochloride (PE); SB-268262, calcitonin gene-related peptide (CGRP) receptor antagonist; sodium nitroprusside (SNP); U46,619 (thromboxane A_2_ analog); verbenol; and, ^13^C-verbenol (Toronto Research Chemicals).

### Animals

Adult male and female C57BL/6J (aka wild type, WT; Strain #000664) and male eNOS-null mice (*Nos3*; 12 wk old; B6.129P2-Nos3tm1Unc/J) were purchased from The Jackson Laboratory (Bar Harbor, ME), or WT and TRPA1-null mice (12–20 wk old; 20–35 g) were used from our in house breeding colony (20). All mice were treated according to the American Physiological Society’s “Guiding Principles in the Care and Use of Animals,” and all protocols were approved by the University of Louisville Institutional Animal Care and Use Committee (IACUC Protocol No. 22204). Mice were housed under pathogen-free conditions in an AAALAC-certified University of Louisville vivarium under controlled temperature, relative humidity, and 12-h:12-h light/dark cycle. Mice were provided water and a standard chow diet ad libitum (Rodent Diet 5010, 4.5% fat by weight, LabDiet; St. Louis, MO) in a cage (up to 5 mice per cage) equipped with a loft, corn cob bedding, and housing enrichment. All mice were euthanized with an overdose of sodium pentobarbital in saline (150 mg/kg body wt ip) followed by exsanguination.

### α-Pinene Exposure

Inhalation exposures were performed in mice only to collect urine for analysis of metabolites of α-pinene. For this purpose, naïve male C57BL/6J mice (*n* = 3) were exposed to HEPA- and charcoal-filtered air for 6 h, and the urine was collected (0–3 h, 3–18 h overnight, in the presence of food). Several days later, the same mice were exposed to α-pinene (1 ppm, 6 h), and the urine was collected in the same manner as after air control exposure. The inhalation exposure was done with a system equipped with a certified permeation tube in a calibrated heating oven (Kin-Tek; LaMarque, TX), as described previously (21). The level of α-pinene was monitored in real-time with an inline electrochemical sensor (CO calibration; MultiRAE Pro, RAE Systems, Burlington, VT) placed upstream of the exposure chamber (30 L capacity; flow 7 LPM). Whole body exposures were done between 7:00 am and 1:00 pm in the absence of food or water (21).

### Urine Collection

Immediately before the filtered air and α-pinene exposures, mice were held and d-glucose-saccharin solution (wt/wt 3.0%–0.125% in drinking water) was touched to their lips and mouth. After each 6-h exposure, each mouse was placed into a separate metabolic cage (Harvard Apparatus; Cambridge, MA) for urine collection within a graduated cylinder held in water of a 4°C water-jacketed organ bath to keep urine cold. During the first 0- to 3-h postexposure urine collection period, mice had access to the glucose:saccharin solution in the cage without food. In the second overnight urine collection (i.e., 3–18+h, O/N), mice were provided both the glucose/saccharin solution and food. Urine samples were centrifuged (1,800 *g*, 5 min; to pellet any feces/food) before being decanted and stored at −80°C until analyses (20).

### Urine Metabolite Analysis

Urinary levels of myrtenol and verbenol, the primary Phase I oxidized metabolites of α-pinene, were measured by gas chromatography-mass spectrometry (GC-MS) as adapted and modified from previous methods (22, 23) for use with an Agilent GC 6890 N/5973 inert MS system with a PAL RSI sample preparation system. Briefly, urine (180 µL) was mixed with sodium phosphate (820 µL; 0.1 M, pH 5.0). with menthol-d_4_ (18 ng) added as an internal standard. β-Glucuronidase-arylsulfatase (5 µL) was added for enzymatic hydrolysis to liberate the phase I metabolites. After incubation at 37°C for 16 h, the enzymatically hydrolyzed urine samples were extracted using a divinylbenzene-polydimethylsiloxane Solid Phase MicroExtraction (SPME) arrow. Then the SPME arrow was exposed to the hot injector of the GC-MS at 250°C. The GC separation was performed with a ZB-5MSplus (30 m × 0.25 mm × 1.0 μm) column (Phenomenex, CA) with a temperature gradient starting at 50°C and ramped up to 260°C. After electron impact (EI) ionization, the analytes were detected in selected ion monitoring (SIM) mode: one SIM was for quantification and at least two SIM were for confirmation of each analyte. MassHunter Workstation Software was used for peak integration, calibration, and quantification. Analytes in urine samples were quantified using peak area ratio based on seven-point standard curves that were run before and after the urine samples. Measured concentrations of α-pinene metabolites were normalized to urinary creatinine (24). Additionally, the total excreted amount of myrtenol and verbenol was estimated by multiplication of the measured urine concentration (in ng/mL) and the total urine volume (in mL) collected over each urine collection period (i.e., 0–3 h, O/N).

### Vasoreactivity of (1R)-(+)-α-Pinene and Oxidized Metabolites in Isolated Murine Aorta and Superior Mesenteric Artery

Only naïve (never exposed) adult male and female C57BL/6J (WT and TRPA1-null) mice were used in isolated blood vessel experiments.

### Isolation and Organ Bath Conditions

After removal and cleaning of aorta and superior mesenteric artery (SMA) in ice-cold buffer, thoracic aorta rings (3–4 mm) were hung on stainless steel hooks in organ baths, whereas SMA rings (2 mm) were hung on tungsten wire in 5-mL organ baths (MultiWire Myograph System 620 M, DMT, Denmark) in Krebs physiological salt solution (PSS) bubbled with 95% O_2_:5% CO_2_ at 37°C. After 10 min without tension, aorta and SMA rings were equilibrated to their respective loading tensions over 30 min (1 g aorta; 0.25 g SMA). All stabilized rings were stimulated with High K^+^ (60 mM) to test for viability, washed 3 times with PSS over 30 min, and reequilibrated to their appropriate resting tension, and stimulated again with High K^+^ (25). PSS for SMA consisted of (in mM) 119 NaCl, 4.7 KCl, 2.0 CaCl_2_, 1.2 MgCl_2_, 1.2 KH_2_PO_4_, 24 NaHCO_3_, and 7.0 glucose at pH 7.4. PSS for aorta consisted of (in mM) 119 NaCl, 4.7 KCl, 1.6 CaCl_2_, 1.2 KH_2_PO_4_, 1.2 MgSO_4_, 25 NaHCO_3_, and 5.5 glucose at pH 7.4. High K^+^ PSS (High K^+^; 60 mM) substituted equimolar K^+^ for Na^+^ in PSS.

### Experimental Series

#### Vasorelaxation.

Parent α-pinene (0.1, 1, 10, 30, 100, 300, and 1,000 µM) or one of the two bicyclic monoterpene alcohol metabolites (myrtenol and verbenol) was added cumulatively to organ baths containing phenylephrine precontracted (PE, 10 µM) aorta and SMA. The efficacy of relaxation was calculated as the % reduction in PE-induced contraction stimulated by ACh. The sensitivity of relaxation was the effective concentration producing 50% response (EC_50_), i.e., cumulative concentration responses normalized to 100% with interpolation of EC_50_ (25). Following exposure to α-pinene or its metabolites, PSS in organ baths was exchanged with fresh PSS (3×) over 30 min, and blood vessels again were precontracted with PE (10 µM). Isometric tension (mN) developed after the first addition of PE was “PE_1_.” The tension (mN) developed after the second addition of PE was “PE_2_.” Contractility (post exposure to either α-pinene or metabolites) was quantified as the ratio of PE_2_ tension to PE_1_ tension (i.e., PE_2_/PE_1_). Notably, the loss of PE_2_ tension (a sign of toxicity), was observed after the addition of 1 mM α-pinene, and resistance to additional contractile agonists was added in succession: U46,619 (thromboxane A_2_ analog) first, and then High K^+^ confirmed toxicity (Table 1; data not shown). Despite minimal contractile tension, a relaxation agonist was added: ACh, first, and, as possible, SNP, an endothelium-independent NO vasorelaxant. The efficacy of relaxation was calculated as the % reduction of the agonist-induced tension. Because of toxicity, concentrations between 0.1 and 300 µM were used for exposure and quantification.

**Table 1.** Efficacy (E_max_), sensitivity (EC_50_), and toxicity following concentration-dependent α-pinene-induced relaxations of PE-precontracted SMA and aorta

Blood Vessel	Control	+l-NAME
*E*_max_, %		
SMA	−91.2 ± 2.6	−93.3 ± 0.8
Aorta	−98.7 ± 1.3	−92.5 ± 4.0
EC_50_, µM		
SMA	72.6 ± 40.4	413.4 ± 45.9*
Aorta	436.5 ± 10.9	481.1 ± 13.0

	PE-induced tension, %1st PE	ACh, %relaxation	SNP, %relaxation
*Toxicity (postincubation with α-pinene)*			
SMA (control)	90.8 ± 14.9	−96.5 ± 2.2	−97.9 ± 0.7
SMA	4.3 ± 1.9*	−9.5 ± 9.5*	−83.3 ± 16.7
Aorta (control)	99.6 ± 8.0	−88.7 ± 3.3	−99.7 ± 0.2
Aorta	5.2 ± 5.2*	0 ± 0*	−100 ± 0

Values are means ± SE; *n* = 3–7 mice per group. All blood vessels were from naïve wild-type male mice. *E*_max_, maximal relaxation; EC_50_, effective concentration producing 50% response; PE, phenylephrine (10 µM); SMA, superior mesenteric artery; l-NAME, *N*^ω^-nitro-l-arginine methyl ester hydrochloride (100 µM); ACh, acetylcholine; SNP, sodium nitroprusside. For comparison of two groups, a paired or an unpaired *t* test (as appropriate) was used. *P < 0.05, treated vs. control (significant difference).

#### Role of the endothelium, nitric oxide synthase, and guanylyl cyclase.

The role of the endothelium in α-pinene-induced vasorelaxation was studied in SMA with intact and injured endothelium. The endothelium was mechanically injured by air perfusion and damage was confirmed by near complete abolition (>95%) of ACh-induced dilation of PE-precontracted SMA (25). To evaluate the role of nitric oxide synthase (NOS), l-NAME (100 µM) was added after the addition of PE (10 µM; 15 min), and after a tension plateau was reached, the precontracted SMA were then relaxed with cumulative concentrations of α-pinene or metabolites (1–300 µM). To assess whether guanylyl cyclase (GC) activity (and formation of cGMP) was involved in vasorelaxation, PE-precontracted SMA were exposed to ODQ (3 µM; 10 min), to inhibit GC, followed by cumulative addition of α-pinene metabolites (0.1–300 µM).

#### Role of TRPA1.

The role of the TRPA1 channel was assessed by incubation of the PE-precontracted blood vessel with a TRPA1 antagonist (A-967079, 1 µM) followed by cumulative addition of either α-pinene or one of its metabolites, myrtenol and verbenol (0.1–300 µM). To confirm the efficacy of the TRPA1 antagonist, the effects of α-pinene or its metabolites also were tested in PE-precontracted SMA of TRPA1-null mice.

As activated TRPA1 in sensory neurons may release CGRP, a known vasodilator, we tested whether a CGRP receptor antagonist (SB-268262, 1 µM) would block part or whole the α-pinene-induced vasorelaxation. As a positive control for receptor antagonist efficacy, we tested whether SB-268262 (1 µM) would prevent CGRP-induced relaxations in PE-precontracted SMA.

### TRPA1 Immunofluorescence

Thin sections (4 μm) of formalin-fixed, paraffin-embedded SMA of WT mice were stained with hematoxylin and eosin or antibodies (TRPA1, isolectin GS-IB4). Images of SMA sections were made using a digital Spot camera mounted on an Olympus microscope. Immunofluorescence microscopy was performed with rabbit polyclonal antibody against TRPA1 (1:200 dilution; Alomone Labs Cat. No. ACC-037, RRID:AB_2040232) and Alexa Fluor 647 goat anti-rabbit secondary antibody (1:400 dilution; Invitrogen; 21244) with or without a blocking peptide of the TRPA1 antibody. To tag endothelial cells, sections were costained with isolectin GS-IB4 Alexa Fluor 594 conjugate (1:200 dilution; Invitrogen; I21413). Briefly, the slides were deparaffinized, and after heat-mediated antigen retrieval (10 mM citrate buffer, pH 6.0, 20 min, 95°C), slides were cooled to RT; incubated with blocking buffer (10% goat serum, 1% BSA, 0.025% triton X-100 in 1× TBS) for 2 h at RT; and, then incubated with either TRPA antibody with or without TRPA1 blocking peptide (Alomone; BLP-CC037; TRPA1 antibody:blocking peptide, 1:1 [μg/μg]; mixed and preincubated for 1 h at RT before adding onto sections) in blocking buffer overnight at 4°C. The slides were incubated with anti-rabbit Alexa Fluor 647 secondary antibody in the dark for 1 h at RT. The slides were washed 3 × 5 min with 1× TBST after each step. Slides were covered with 4′,6-diamidino-2-phenylindole (DAPI) containing Slow Fade Gold antifade reagent (Invitrogen; S36938), and fluorescence was visualized on a Nikon eclipse Ti fluorescence Microscope using NIS-Elements (×200 magnification).

### Statistical Analyses

Data are expressed as means ± SE. When comparing two concentration-dependent response curves, a two-way ANOVA with repeated measures and Bonferroni all pairwise post hoc test was used, and when comparing multiple groups versus a control a one-way ANOVA with repeated measures and Bonferroni post hoc test was used (SigmaPlot, ver. 12). As vascular toxicity was detected after addition of 1 mM α-pinene (Table 1), all analyses were performed on responses up to 300 μM. Statistical significance was assumed where *P* < 0.05.

## RESULTS

### Exposure of Mice to α-Pinene Increased Urinary Excretion of Two Oxidized Metabolites: Myrtenol and Verbenol

**Figure 1.**
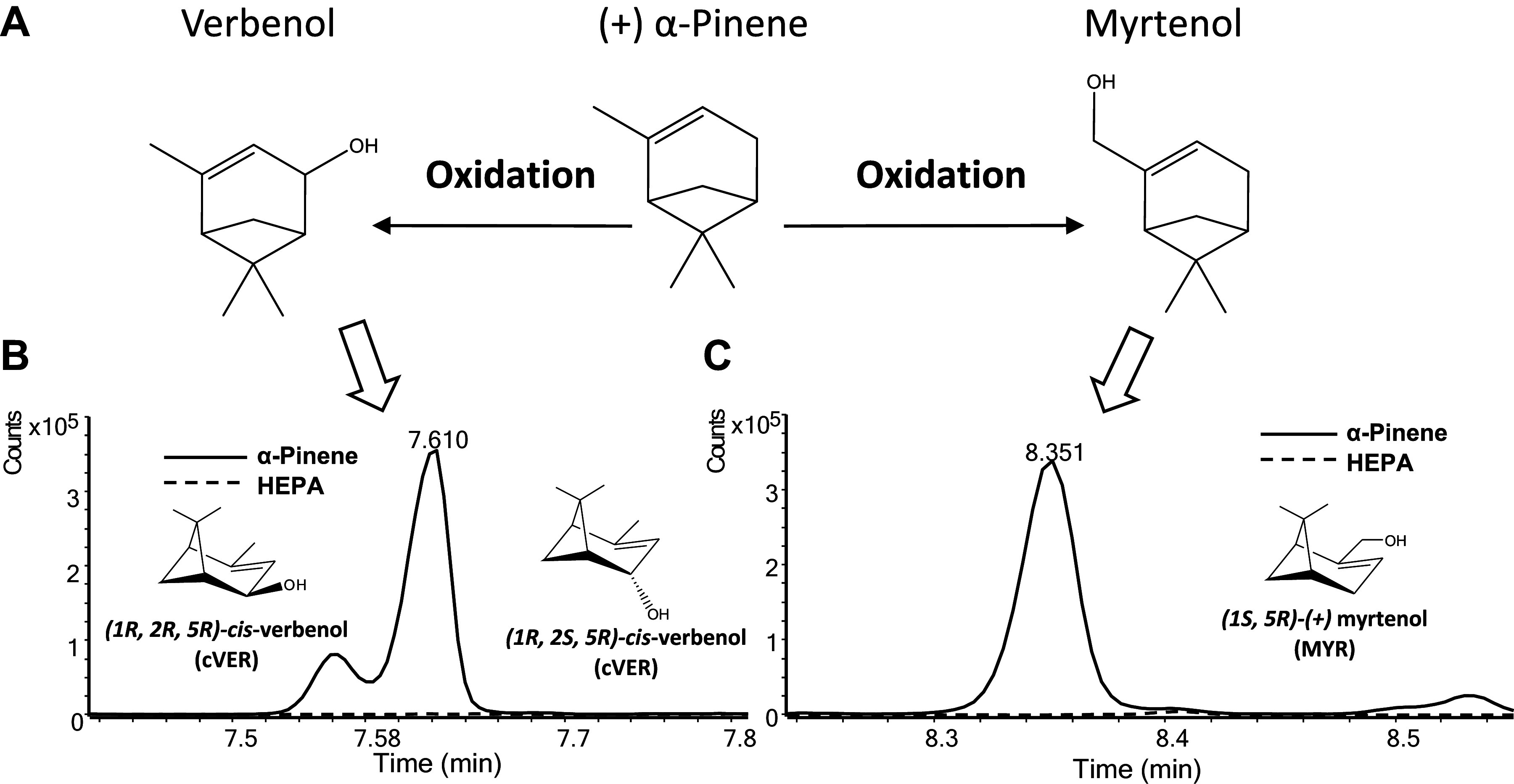
Metabolism of inhaled α-pinene in mice. *A*: chemical structures of α-pinene and two oxidized α-pinene metabolites: verbenol and myrtenol. Two oxidation metabolites of α-pinene were identified and quantified as *cis*- and *trans*-verbenol isomers [(1*R*,2*R*,5*R*)-verbenol and (1 *R*,2*S*,5*R*)-verbenol] (*B*) and myrtenol [(1*S*,5*R*)-(+)-myrtenol] using GC-MS (*C*). GC-MS analyses were done using urine collected from adult male C57BL/6J mice (*n* = 3) after exposure to HEPA- and charcoal-filtered air (6 h) and after single exposure to α-pinene (1 ppm; 6 h).

Following α-pinene inhalation exposure (1 ppm, 6 h), two urinary oxidized metabolites were identified and quantified by GC-MS (Fig. 1, *A–C*). Baseline urine excretion of myrtenol or verbenol was undetectable in HEPA-filtered air-exposed control mice (6 h) indicating that these metabolites were undetectable in the urine of laboratory C57BL/6 (WT) mice housed with standard corn cob bedding and a normal laboratory chow-based diet (Fig. 1, *B* and *C*). Subsequently, the levels of urinary myrtenol and verbenol (in μg) excreted from WT mice increased dramatically at 0–3 h after a 6-h inhalation exposure to α-pinene (1 ppm). Two oxidized alcohol metabolites of (+)-α-pinene were identified and quantified as *cis*- and *trans*-verbenol [(1*R*,2*R*,5*R*)-verbenol, (1 *R*,2*S*,5*R*)-verbenol; 3,402 ± 908 ng/mL, *n* = 3 mice; Fig. 1*B*] and myrtenol [(1*S*,5*R*)-(+)-myrtenol; 444 ± 117 ng/mL, *n* = 3 mice; Fig. 1*C*] by GC-MS. From the quantification of urinary levels, we estimated that the blood level of these metabolites (combined) may exceed 10 μM during inhalation exposure. To estimate a blood level, the total excreted myrtenol and verbenol metabolite load (3,846 ng/mL urine) was multiplied by the total amount of urine collected in 3 h (1 mL); divided by the total blood volume of a 25 g mouse (est. 2 mL); and, then divided by the metabolite MW (152.23) to estimate a blood molarity of [12.6 μM]. As these metabolites are further metabolized, their blood levels are, of course, constantly in flux throughout and after the exposure.

### Concentration-Dependent Effects

**Figure 2.**
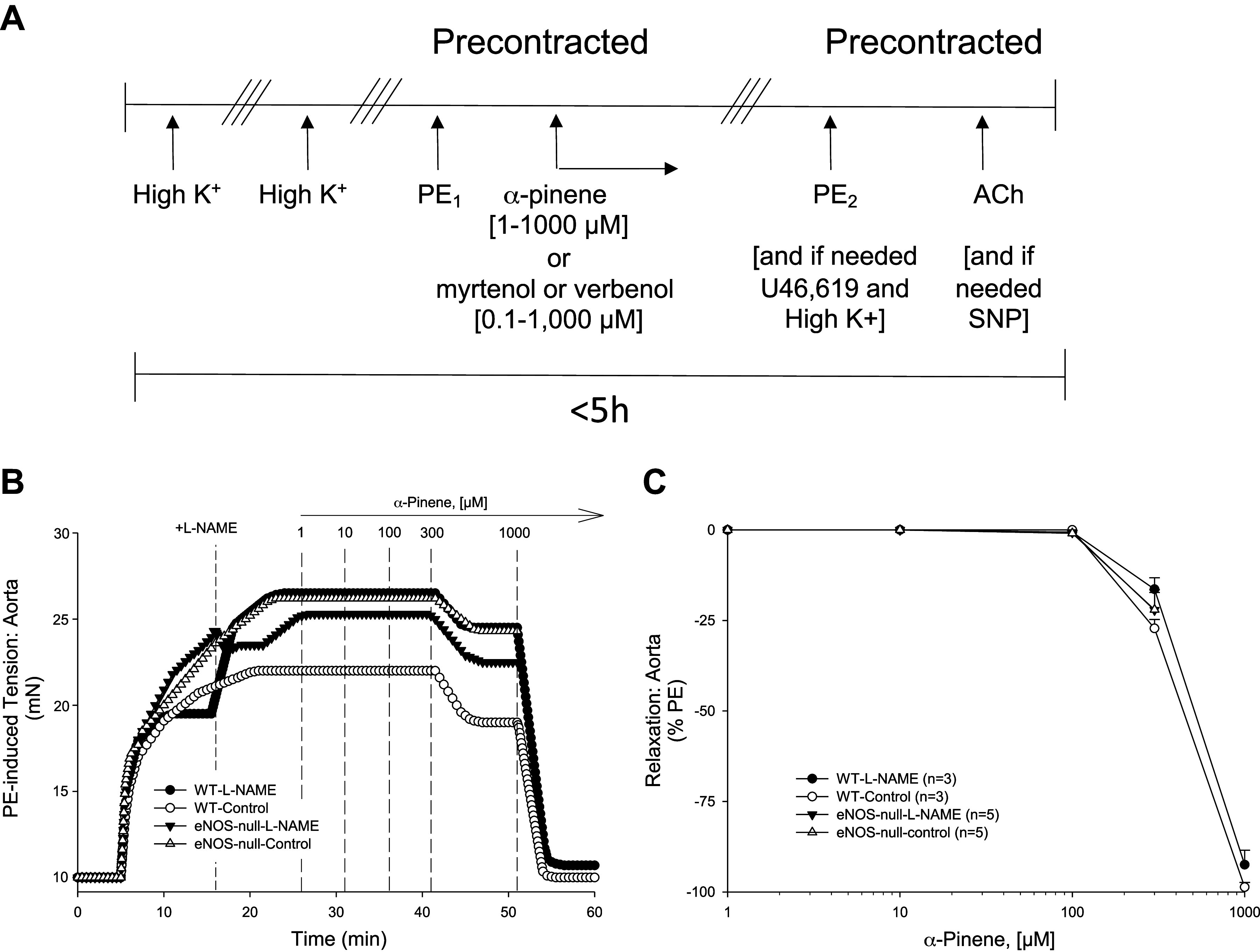
Testing of α-pinene-induced relaxation in C57BL/6J blood vessels. *A*: protocol for testing of α-pinene and its metabolites in precontracted blood vessels. Aorta and superior mesenteric artery (SMA) were stimulated with 60 mM High K^+^ followed by washout [i.e., 3 exchanges of fresh physiological salt solution (PSS);///], and the process repeated once more. Blood vessels were then precontracted with 10 µM phenylephrine (PE_1_) followed by cumulative, concentration-dependent addition of α-pinene or its metabolites (myrtenol, verbenol) (in the range of 0.1–1,000 µM). After 3× washout and to assess potential vasotoxicity, blood vessels were stimulated with PE (PE_2_), and then 100 nM U46619, and then High K^+^, as warranted based on PE_2_, followed by 10 µM acetylcholine (ACh) and then 100 µM sodium nitroprusside (SNP) to assess relaxation capacity. Total time of in vitro experiments was <5 h. Representative traces (*B*) and summary data (*C*) of 1–300 µM α-pinene-stimulated relaxation of PE-precontracted aorta of wild-type (WT) and endothelial nitric oxide synthase (eNOS)-null mice in the absence (control) and in the presence of 100 µM *N*^ω^-nitro-l-arginine methyl ester (l-NAME). Values are means ± SE; *n*, 3–5 mice per group. For comparison of two concentration-dependent response curves, a two-way ANOVA with repeated measures and Bonferroni all pairwise post hoc test was used.

To test for vasoactive effects of parent α-pinene, we isolated blood vessels from naïve mice (never exposed). In PE-precontracted aorta and SMA, cumulative addition of (+) α-pinene (from 1 to 1,000 μM) induced robust, concentration-dependent relaxations of >90% reduction in tension (Fig. 2, Table 1). However, in aorta, a significantly less sensitive, concentration-dependent relaxation was accompanied by a curious “rebound contraction” at 1 mM, which then was followed by a slower and longer-lasting relaxation. Following 3 organ bath buffer exchanges (see Fig. 2*A*), both aorta and SMA were refractory to addition of each of 3 different, sequentially added contractile agents: PE (10 μM), U46,619 (0.1 μM), and High K^+^ (60 mM) indicating that exposure of aorta and SMA to α-pinene at 1 mM led to severe vasotoxicity, perhaps irreversible (Table 1). Thus, because of this toxicity and despite the robust relaxation at 1 mM, α-pinene concentrations ≤300 μM were used in all subsequent experiments.

**Figure 3.**
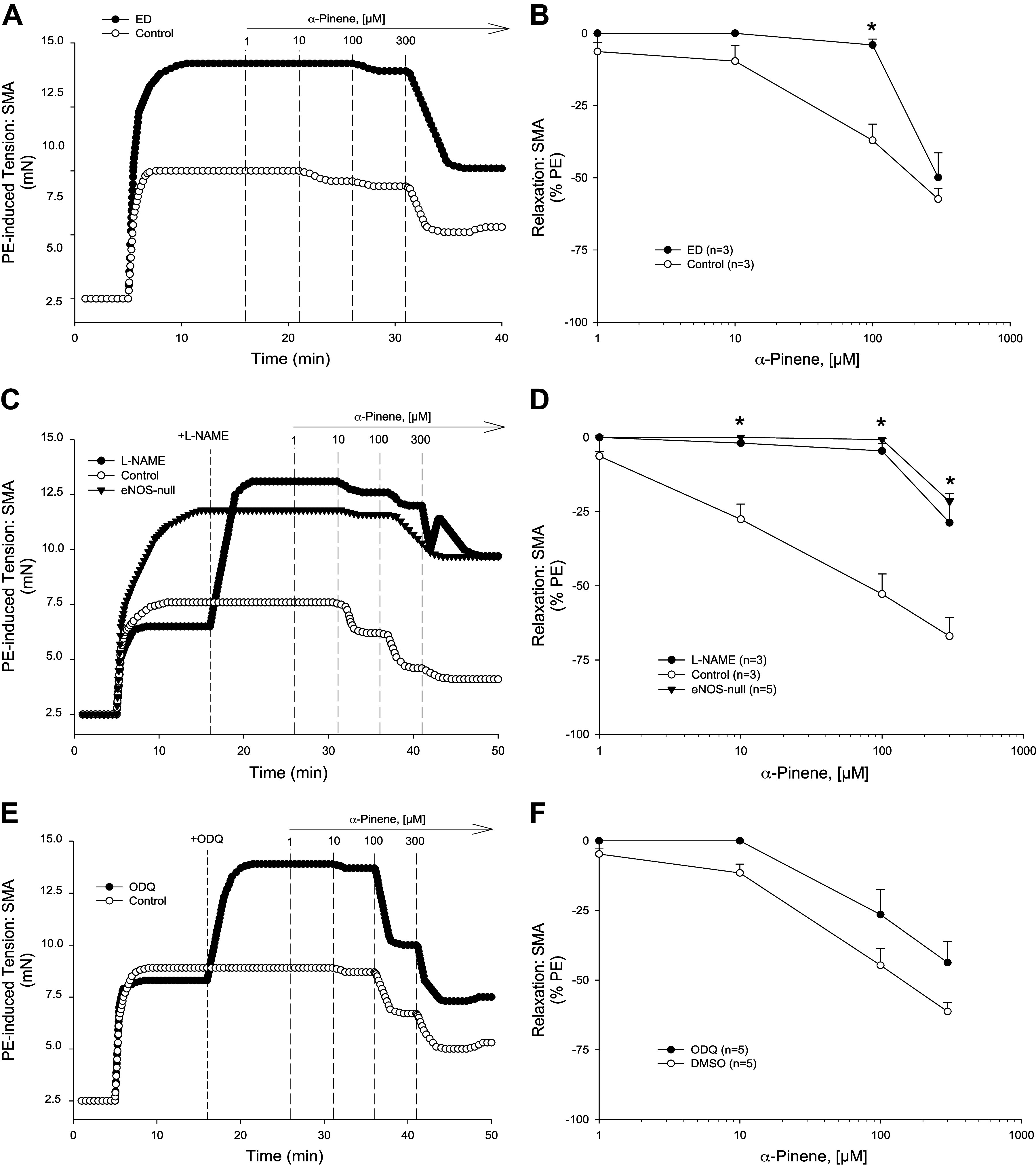
α-Pinene induced relaxations in precontracted C57BL/6J superior mesenteric artery (SMA). Representative traces (*A*) and summarized data (*B*) of 1–300 µM α-pinene-stimulated relaxation of 10 µM phenylephrine (PE)-precontracted wild-type (WT) SMA without and with mechanically induced endothelial dysfunction (ED; *n* = 3, 3 mice, respectively). Representative traces (*C*) and summarized data (*D*) of 1–300 µM α-pinene-stimulated relaxation of 10 µM PE-precontracted WT SMA without (control) and with 100 µM *N*^ω^-nitro-l-arginine methyl ester (l-NAME) or in SMA from endothelial nitric oxide synthase (eNOS)-null mice (*n* = 3, 3, 5 mice, respectively). Representative traces (*E*) and summarized data (*F*) of 1–300 µM α-pinene-stimulated relaxation of 10 µM PE-precontracted WT SMA without (control; DMSO) and with 3 µM 1 h-[1,2,4]oxadiazolo[4,3-a]quinoxalin-1-one (ODQ) (*n* = 5, 5 mice, respectively). Values are means ± SE; *n*, 3–5 mice per group. For comparison of two concentration-dependent response curves, a two-way ANOVA with repeated measures and Bonferroni all pairwise post hoc test was used. *P < 0.05, treatment vs. control.

Overall, α-pinene induced robust, concentration-dependent relaxations in both aorta (Fig. 2, *B* and *C*) and SMA (Fig. 3). However, in SMA there was a rapid onset of relaxation at 1–10 μM that continued up to 300 μM (Fig. 3, *A* and *B*). The SMA was significantly more sensitive (6×) than the aorta to α-pinene-induced (0.1–300 μM) relaxation (Table 1, Table 3). Isolated naïve female SMA were less sensitive (3.5×) than naïve male SMA to α-pinene (Table 3). Because the male SMA was more sensitive than female SMA, it was used for all subsequent mechanistic experiments.

To investigate the mechanisms of α-pinene, we first studied the role of the endothelium. After mechanical endothelium disruption by air perfusion (5 min; >90% loss of ACh-induced relaxation), SMA relaxation to α-pinene was significantly depressed and right-shifted (Fig. 3, *A* and *B*, and Tables 2 and 3). To focus on specific endothelial cell targets, we tested α-pinene-induced relaxations in SMA of male eNOS-null mice and after l-NAME pretreatment (Fig. 3, *C* and *D*). l-NAME pretreatment and eNOS deletion in SMA significantly suppressed and shifted relaxation of α-pinene (Tables 2 and 3). Notably, neither l-NAME pretreatment nor eNOS deletion had any effect on α-pinene-induced relaxations in aorta (Fig. 2, *B* and *C*). These data indicated a strong dependence of α-pinene-induced relaxation on endothelium-derived NO (eNOS) in SMA, but not in the aorta. Because NO stimulates soluble guanylyl cyclase (GC) in vascular smooth muscle cells (VSMC) to generate cGMP and activate PKG-mediated relaxation, we tested whether pretreatment with an inhibitor of GC, ODQ (1 h-[1,2,4]oxadiazolo[4,3-a]quinoxalin-1-one), would affect α-pinene-induced relaxation. Indeed, ODQ pretreatment significantly shifted and attenuated the α-pinene-induced relaxation in SMA further supporting the role of NO in relaxation (Fig. 3, *E* and *F*, and Tables 2 and 3).

**Table 2.** Efficacy (E_max_) of 300 µM α-pinene-, myrtenol-, and verbenol-induced relaxations in PE-precontracted SMA (male and female) with and without inhibitor treatments or SMA of transgenic mice

SMA		*E*_max_, %	Change, Absolute%
	*n*		
α-Pinene			
Control	10	−66.6 ± 3.3	ND
+ED	3	−49.9 ± 8.6*	−15
+l-NAME	3	−28.7 ± 8.2*	−36
eNOS-null	5	−21.7 ± 2.8*	−43
+ODQ	5	−43.7 ± 7.6*	−21
+A-967079	4	−63.0 ± 6.8	ND
TRPA1-null	3	−33.7 ± 3.0*	−31
WT (f)	4	−48.4 ± 12.4	−16
SB-268262	3	−71.6 ± 6.5	+7
Myrtenol			
Control	7	−64.4 ± 2.0	ND
+ED	3	−44.5 ± 7.0*	−20
+l-NAME	3	−36.1 ± 4.8*	−28
eNOS-null	5	−36.5 ± 2.0*	−28
+ODQ	4	−37.5 ± 3.0*	−27
+A-967079	4	−68.8 ± 3.7	ND
TRPA1-null	3	−65.0 ± 5.0	ND
WT (f)	4	−63.9 ± 5.5	ND
Verbenol			
Control	5	−67.0 ± 3.1	ND
+ED	3	−47.9 ± 2.6*	−18
+l-NAME	3	−41.2 ± 7.3*	−26
eNOS-null	5	−27.7 ± 4.4*	−40
+ODQ	3	−34.6 ± 3.5*	−33
+A-967079	4	−68.7 ± 2.0	ND
TRPA1-null	3	−58.2 ± 3.8	−9
WT (f)	4	−55.7 ± 4.1	−12

Values are means ± SE; *n* = 3–10 mice per group. All superior mesenteric arteries (SMAs) were from naïve male mice, except where naïve female (f). *E*_max_, maximal relaxation; PE, phenylephrine (10 µM); ED, endothelial dysfunction by mechanical air perfusion; l-NAME, *N*^ω^-nitro-l-arginine methyl ester hydrochloride (100 µM); eNOS, endothelial nitric oxide synthase; ODQ, 1 h-[1,2,4]oxadiazolo[4,3-a]quinoxalin-1-one (3 µM); TRPA1, transient receptor potential ankyrin-1 (1 µM); A-967079, TRPA1 agonist; WT, wild-type; ND, no difference. For comparison of multiple groups, 1-way ANOVA with Bonferroni posttest was used. *P < 0.05; treatment vs. control.

**Table 3.** Sensitivity (EC_50_) of 0.1–300 µM α-pinene-, myrtenol-, and verbenol-induced relaxations in PE-precontracted murine SMA in the presence and absence of treatments or SMA of transgenic mice

SMA		EC_50_, µM	Change, ×
	*n*		
α-Pinene			
Control	10	33.1 ± 8.2	ND
+ED	3	166.1 ± 3.8*	5
+l-NAME	3	157.5 ± 10.3*	5
eNOS-null	5	169.8 ± 1.9*	5
+ODQ	5	89.8 ± 25.2*	2.5
+A-967079	4	38.3 ± 15.3	ND
TRPA1-null	3	135.5 ± 12.9*	4
WT (f)	4	123.9 ± 18.9*	3.5
SB-268262	3	11.8 ± 4.1	ND
Myrtenol			
Control	7	34.0 ± 6.1	ND
+ED	3	107.2 ± 13.1*	3
+l-NAME	5	118.4 ± 13.5*	3.5
eNOS-null	5	141.9 ± 2.0*	4
+ODQ	4	119.7 ± 7.7*	3.5
+A-967079	4	52.1 ± 7.5	1.5
TRPA1-null	3	47.5 ± 7.2	ND
WT (f)	4	50.8 ± 31.2	ND
Verbenol			
Control	5	39.7 ± 7.7	ND
+ED	3	113.7 ± 5.6*	3
+l-NAME	3	106.4 ± 21.5*	2.5
eNOS-null	5	135.7 ± 4.6*	4
+ODQ	3	126.9 ± 9.8*	3
+A-967079	4	51.6 ± 10.7	ND
TRPA1-null	3	75.9 ± 7.8	1.5
WT (f)	4	52.0 ± 13.1	ND

Values are means ± SE; *n* = 3–10 mice per group. All superior mesenteric arteries (SMAs) were from naïve male mice, except where naïve female (f). *E*_max_, maximal relaxation; PE, phenylephrine (10 µM); ED, endothelial dysfunction induced by 5-min mechanical air perfusion; l-NAME, *N*^ω^-nitro-l-arginine methyl ester hydrochloride (100 µM); eNOS, endothelial nitric oxide synthase; ODQ, 1 h-[1,2,4]oxadiazolo[4,3-a]quinoxalin-1-one (3 µM); TRPA1, transient receptor potential ankyrin-1 (1 µM); A-967079, TRPA1 agonist; WT, wild-type; ND, no difference. For comparison of multiple groups, 1-way ANOVA with Bonferroni posttest was used. *P < 0.05; treatment vs. respective control (α-pinene, myrtenol, verbenol).

**Figure 4.**
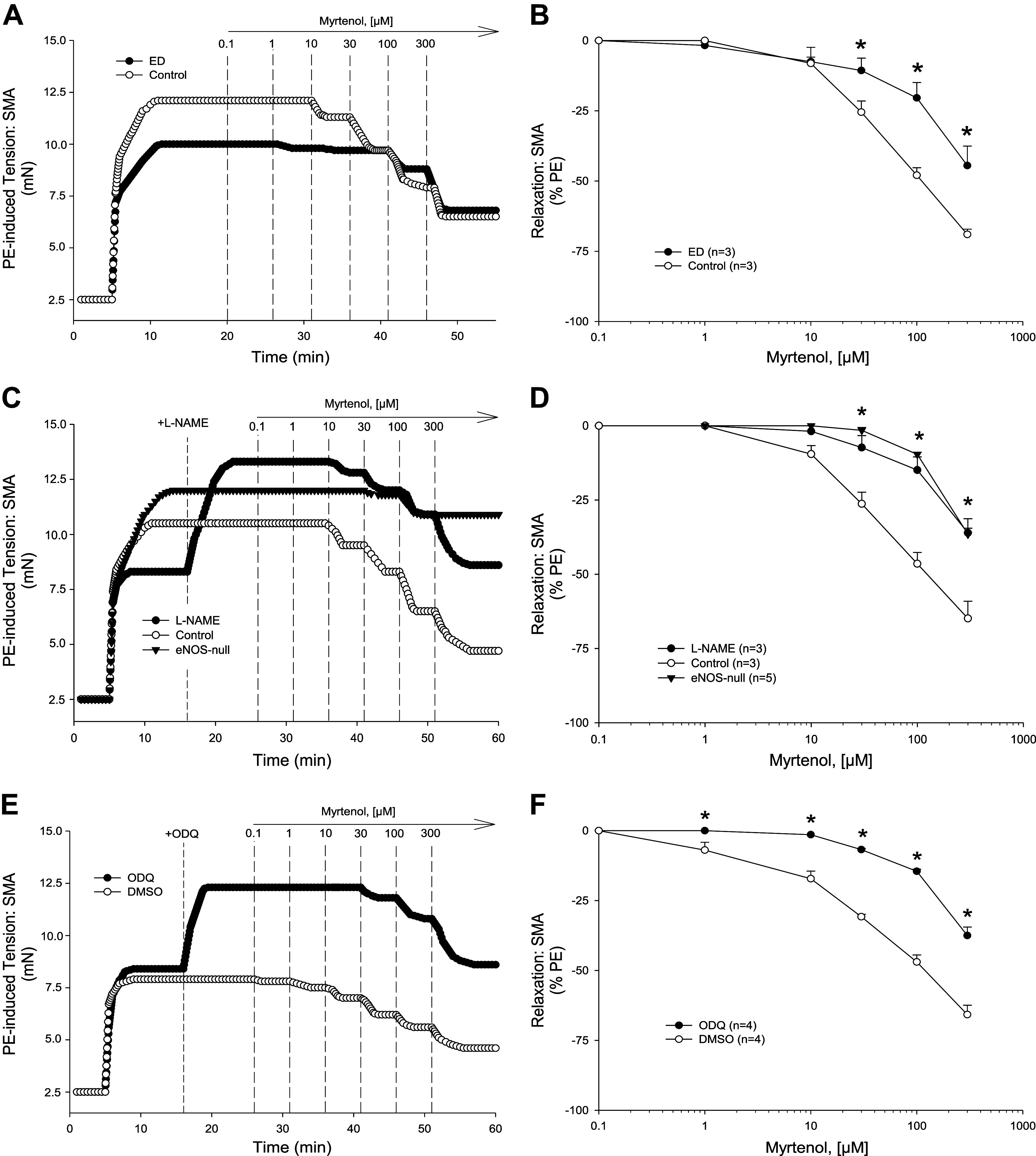
Mechanisms of myrtenol-induced relaxation in C57BL/6J superior mesenteric artery (SMA). Representative traces (*A*) and summarized data (*B*) of 1–300 µM myrtenol-stimulated relaxation of 10 µM phenylephrine (PE)-precontracted wild-type (WT) SMA without and with mechanically induced endothelial dysfunction (ED; *n* = 3, 3 mice, respectively). Representative traces (*C*) and summarized data (*D*) of 1–300 µM myrtenol-stimulated relaxation of 10 µM PE-precontracted WT SMA without (control) and with 100 µM *N*^ω^-nitro-l-arginine methyl ester (l-NAME) or in SMA from endothelial nitric oxide synthase (eNOS)-null mice (*n* = 3, 3, 5 mice, respectively). Representative traces (*E*) and summarized data (*F*) of 1–300 µM myrtenol-stimulated relaxation of 10 µM PE-precontracted WT SMA without [control; dimethyl sulfoxide (DMSO)] and with 3 µM 1 h-[1,2,4]oxadiazolo[4,3-a]quinoxalin-1-one (ODQ) (*n* = 4, 4 mice, respectively). Values are means ± SE of 3–5 mice per group. For comparison of two concentration-dependent response curves, a two-way ANOVA with repeated measures and Bonferroni all pairwise post hoc test was used. *P < 0.05, treatment vs. control.

**Figure 5.**
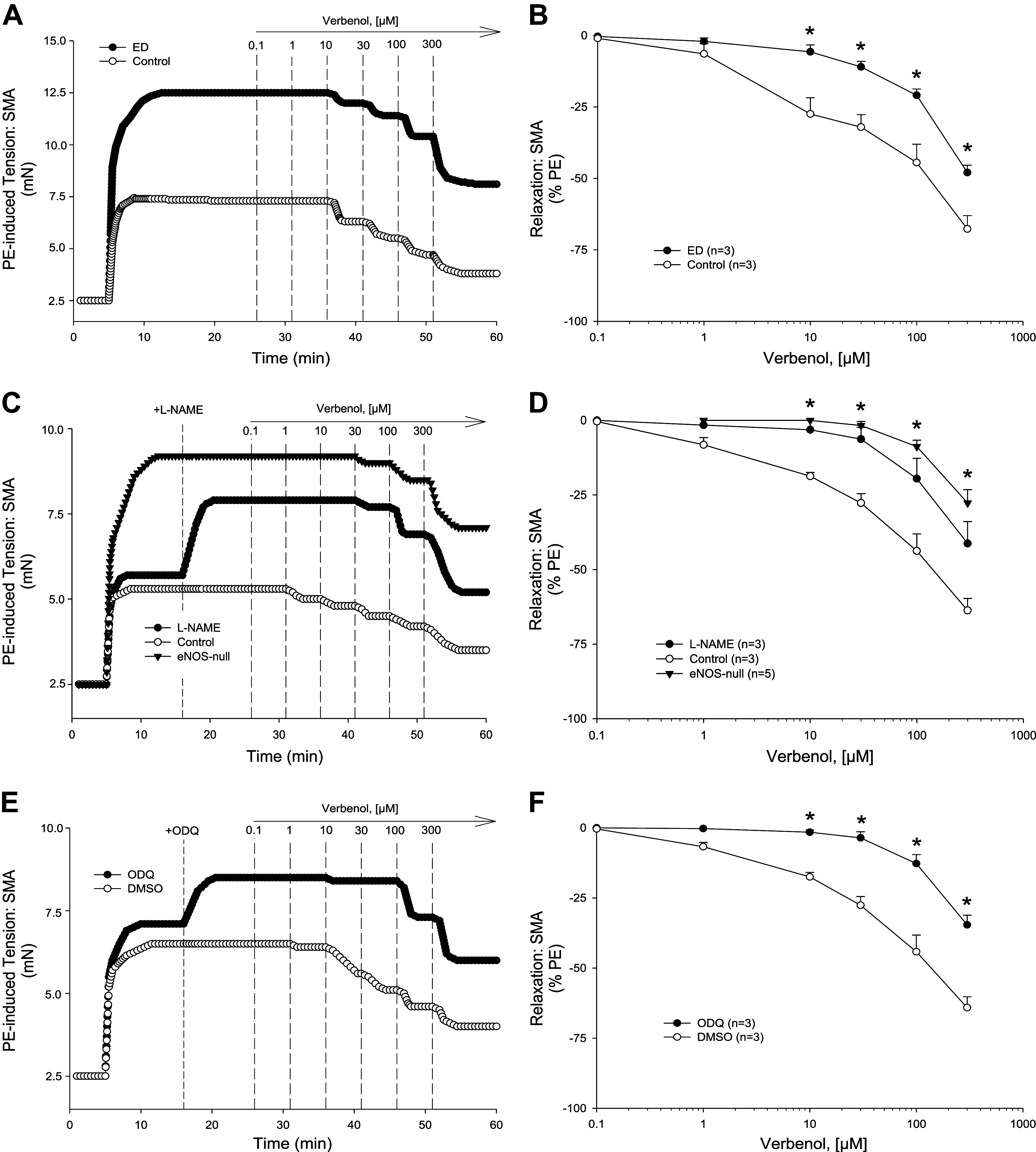
Mechanisms of verbenol-induced relaxation in C57BL/6J superior mesenteric artery (SMA). Representative traces (*A*) and summarized data (*B*) of 1–300 µM verbenol-stimulated relaxation of 10 µM phenylephrine (PE)-precontracted wild-type (WT) SMA without and with mechanically induced endothelial dysfunction (ED; *n* = 3, 3 mice, respectively). Representative traces (*C*) and summarized data (*D*) of 1–300 µM verbenol-stimulated relaxation of 10 µM PE-precontracted WT SMA without (control) and with *N*^ω^-nitro-l-arginine methyl ester (l-NAME) (100 µM) or in SMA from endothelial nitric oxide synthase (eNOS)-null mice (*n* = 3, 3, 5 mice, respectively). Representative traces (*E*) and summarized data (*F*) of 1–300 µM verbenol-stimulated relaxation of 10 µM PE-precontracted WT SMA without [control; dimethyl sulfoxide (DMSO)] and with 3 µM 1 h-[1,2,4]oxadiazolo[4,3-a]quinoxalin-1-one (ODQ) (*n* = 3, 3 mice, respectively). Values are means ± SE; *n*, 3–5 mice per group. For comparison of two concentration-dependent response curves, a two-way ANOVA with repeated measures and Bonferroni all pairwise post hoc test was used. *P < 0.05, treatment vs. control.

We then tested whether the α-pinene oxidation metabolites that we identified in Fig. 1, myrtenol and verbenol, could induce relaxation in precontracted SMA. Both metabolites induced robust relaxation in SMA that were equally efficacious and potent as parent α-pinene (Figs. 4 and 5, and Tables 2 and 3).

### Mechanisms of Myrtenol- and Verbenol-Induced Relaxation in SMA

To understand how myrtenol (primary alcohol) and verbenol (secondary alcohol) induced relaxation, the specific mechanisms of action of each compound were determined in PE-precontracted SMA. Between 0.1 and 300 μM, both myrtenol (Fig. 4)- and verbenol (Fig. 5)-induced relaxations that were significantly inhibited in PE-precontracted SMA by *1*) mechanically disrupted endothelium (Figs. 4, *A* and *B*, and 5, *A* and *B*, and Tables 2 and 3), *2*) presence of l-NAME and absence of eNOS (Figs. 4, *C* and *D*, and 5, *C* and *D*, and Tables 2 and 3), and *3*) ODQ treatment, a GC inhibitor (Figs. 4, *E* and *F*, and 5, *E* and *F*, and Tables 2 and 3). Results of these tests showed a strong, and shared dependence of both myrtenol- and verbenol-induced relaxations in SMA on endothelium-derived NO as was observed with parent α-pinene. Following organ bath buffer exchanges after myrtenol and verbenol (see Fig. 2*A*), isolated SMA of male and female mice were still responsive to PE (10 μM; approximately 90% and 80% tension, respectively, of the first PE-induced contraction: PE_2_/PE_1_). Following exposure of SMA to either metabolite, there was no detectable loss of endothelial function (measured as ACh-induced relaxation) in PE-precontracted SMA (ACh %decrease in PE-tension: male, −95–96%; female, −93–96%) indicating no vasotoxicity as observed after use of α-pinene at 1 mM (Fig. 2, Table 1).

### Role of TRPA1 Channel in α-Pinene-Induced Relaxation

**Figure 6.**
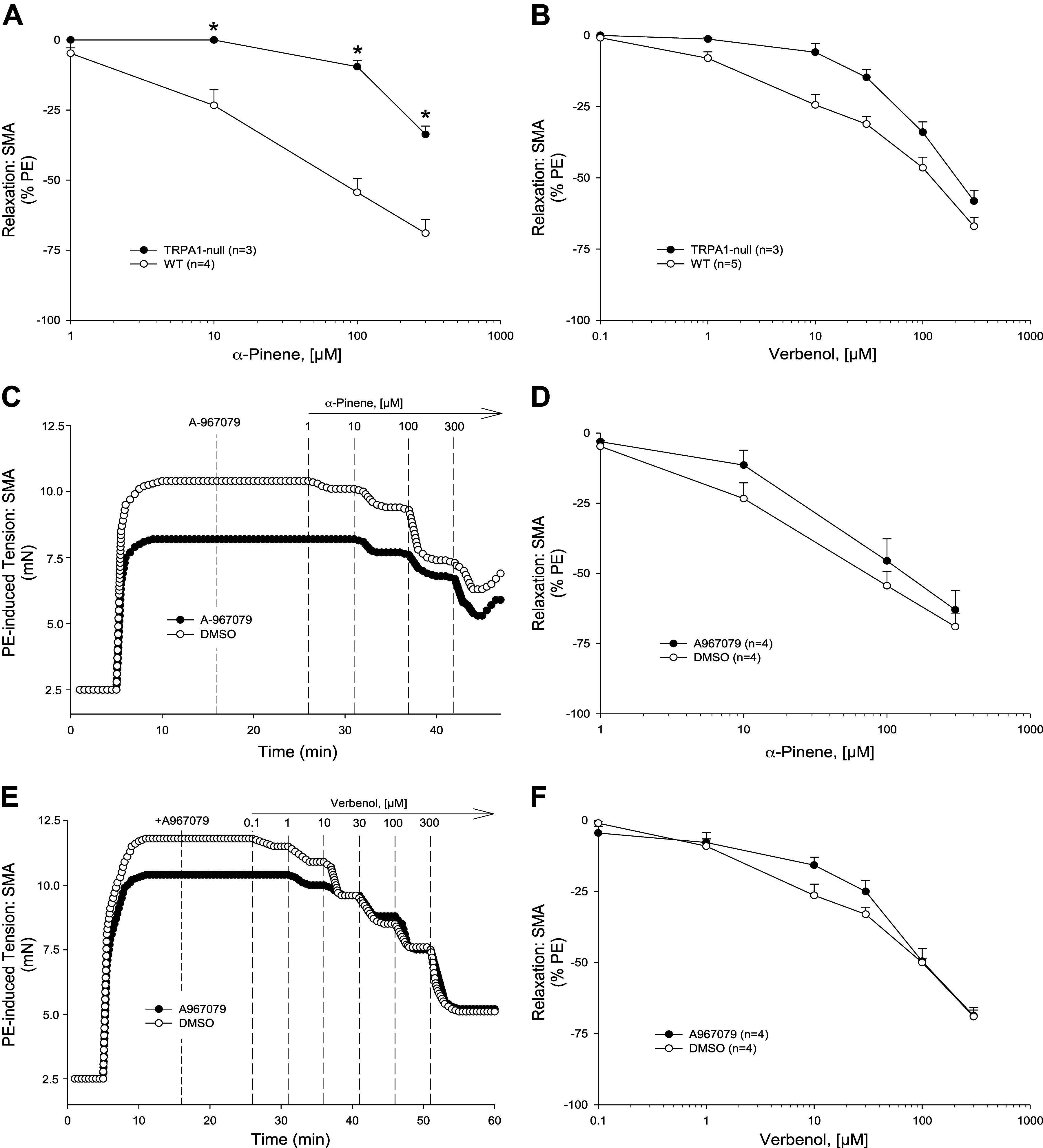
Role of the transient receptor potential ankyrin-1 (TRPA1) channel in α-pinene- and verbenol-induced relaxations in C57BL/6J superior mesenteric artery (SMA). Summary data of α-pinene (*A*) and its metabolite verbenol (*B*) stimulated relaxations of phenylephrine (PE)-precontracted SMA of wild-type (WT) and TRPA-null mice. Representative traces (*C*) and summarized data (*D*) of 1–300 µM α-pinene-stimulated relaxation of 10 µM PE-precontracted WT SMA without [control, plus dimethyl sulfoxide (DMSO)] and with TRPA1 receptor antagonist, A-967079 (1 µM) (*n* = 4, 4 mice, respectively). Representative traces (*E*) and summarized data (*F*) of 0.1–300 µM verbenol-stimulated relaxation of 10 µM PE-precontracted WT SMA without (control, DMSO) and with TRPA1 receptor antagonist, A-967079 (1 µM) (*n* = 4, 4 mice, respectively). Values are means ± SE; *n*, of 3or 4 mice per group. For comparison of two concentration-dependent response curves, a two-way ANOVA with repeated measures and Bonferroni all pairwise post hoc test was used. *P < 0.05, treatment vs. control.

**Figure 7.**
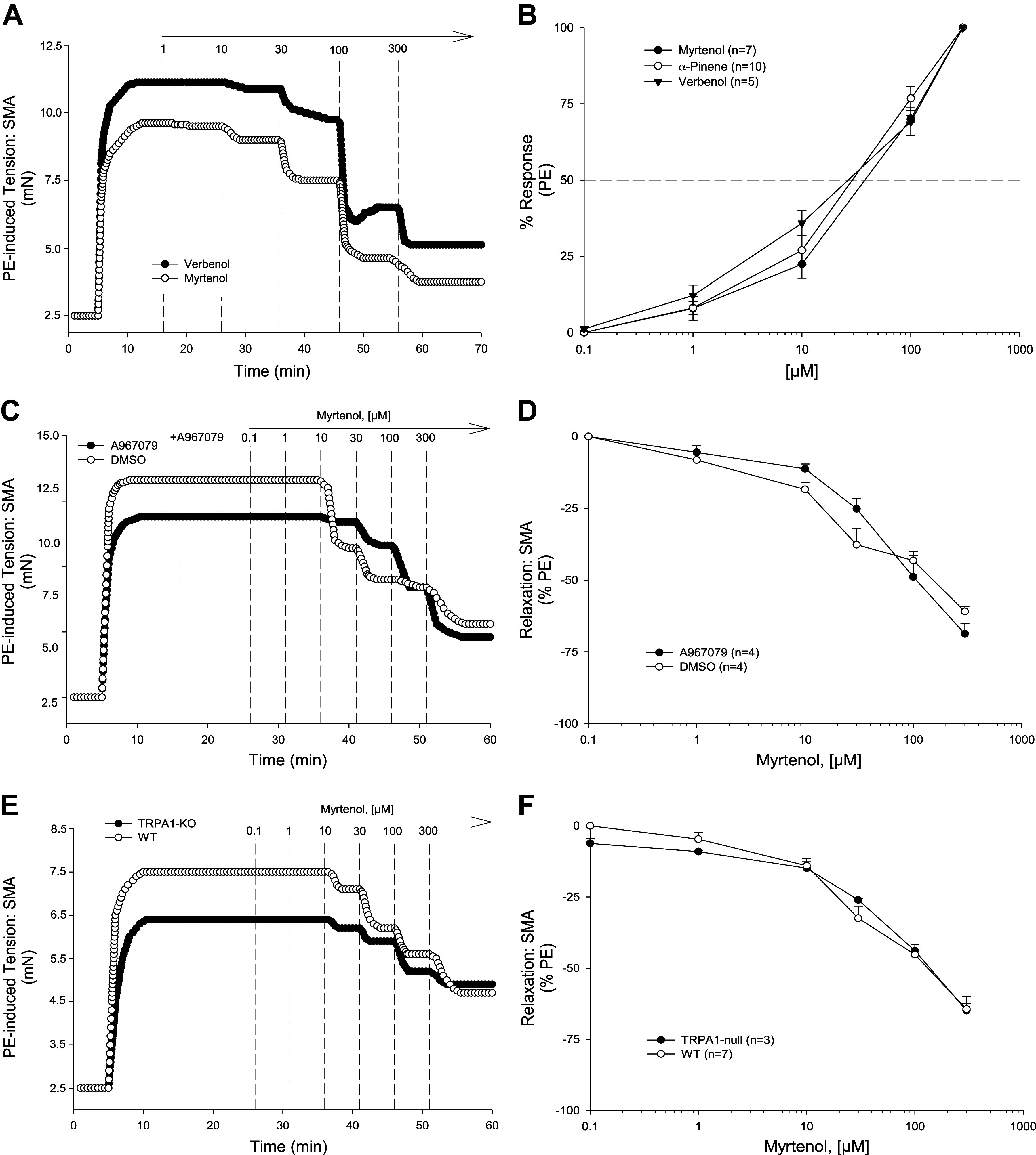
Mechanisms of myrtenol- and verbenol-induced relaxation in C57BL/6J superior mesenteric artery (SMA). Representative traces (*A*) and normalized data (*B*) of myrtenol- and verbenol-stimulated (0.1–300 µM) relaxation of 10 µM phenylephrine (PE)-precontracted SMA (*n* = 3,3 C57BL/6J mice) compared with α-pinene-induced relaxation (*B* only). Representative traces (*C*) and summarized data (*D*) of 1–300 µM myrtenol-stimulated relaxation of 10 µM PE-precontracted wild-type (WT) SMA without [control; dimethyl sulfoxide (DMSO)] and with transient receptor potential ankyrin-1 (TRPA1) inhibitor, A-967079 (1 µM) (*n* = 4, 4 mice, respectively). Representative traces (*E*) and summarized data (*F*) of myrtenol-stimulated relaxations of PE-precontracted SMA of WT and TRPA-null mice (*n* = 7, 3 mice, respectively). Values are means ± SE; *n*, 3–7 mice per group. For comparison of two concentration-dependent response curves, a two-way ANOVA with repeated measures and Bonferroni all pairwise post hoc test was used.

Because we have shown that agonists of TRPA1 also stimulate endothelium- and NO-dependent relaxations in SMA (25, 26), we tested whether the TRPA1 channel contributed to α-pinene-, myrtenol-, and verbenol-induced relaxations in SMA. Notably, SMA of male TRPA1-null mice had a significantly rightward-shifted relaxation response to α-pinene (Fig. 6*A*, Table 3). The response in SMA of male TRPA1-null mice was insignificantly shifted with verbenol (Fig. 6*B*, Table 3), and no shift at all was observed in TRPA1-null SMA with myrtenol (Fig. 7, *E* and *F*, and Table 3) indicating that the absence of TRPA1 inhibited the most sensitive portion of the α-pinene-induced SMA relaxation but not of metabolites (Table 3). Moreover, these data support that the metabolites of α-pinene may be responsible for the majority of the α-pinene-induced relaxation in SMA, yet not via activation of TRPA1 – a novel finding (Tables 2 and 3; Graphical abstract). Inconsistent with our previously published findings, however, the TRPA1 antagonist, A-967079 (1 µM), did not significantly inhibit α-pinene-, myrtenol-, or verbenol-induced relaxations in PE-contracted SMA (Figs. 6, *C*–*F*, and 7, *C* and *D*, and Tables 2 and 3).

**Figure 8.**
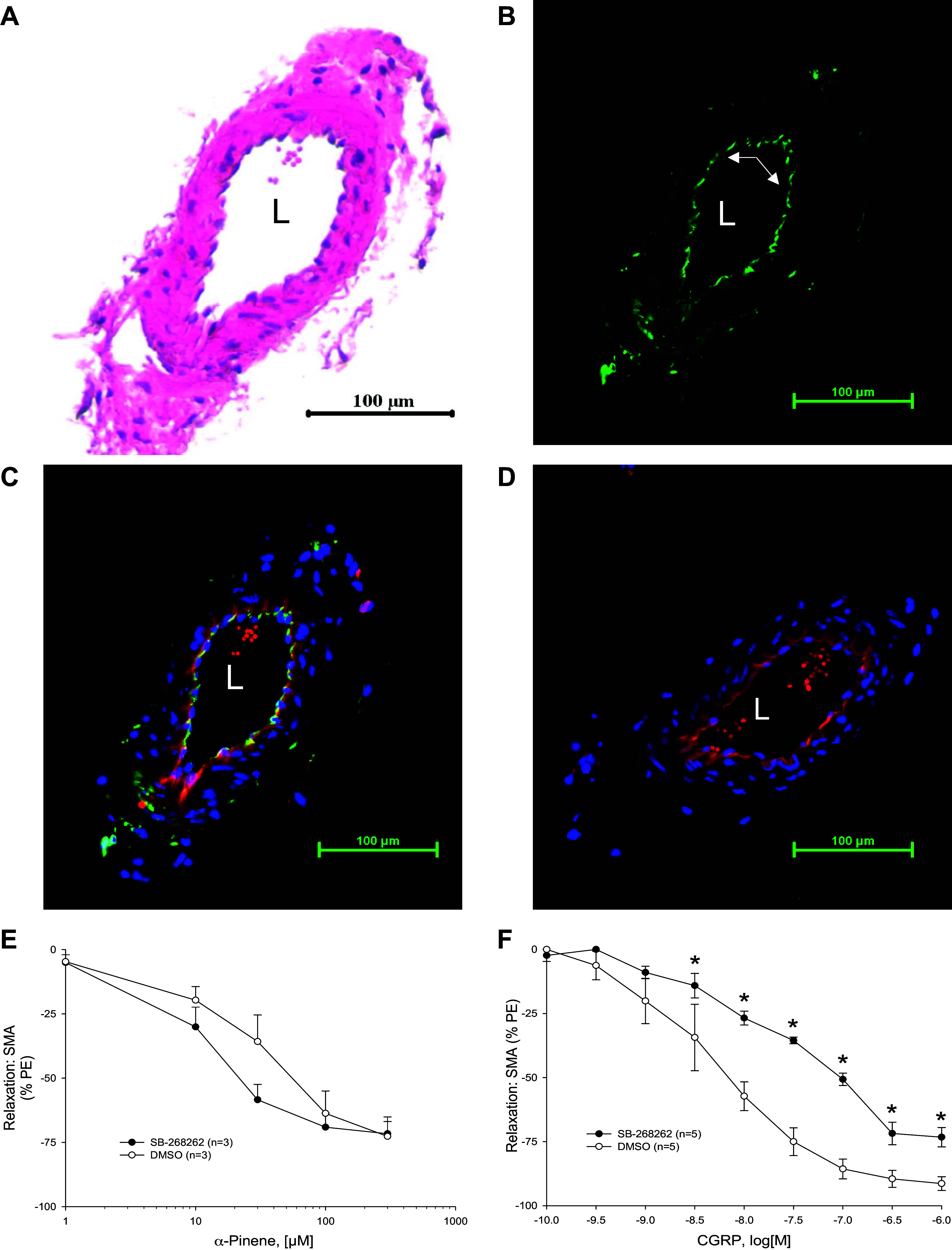
Immunofluorescent localization of transient receptor potential ankyrin-1 (TRPA1) in C57BL/6J superior mesenteric artery (SMA). Formalin-fixed, paraffin-embedded sections of murine male wild-type (WT) SMA were stained with hematoxylin and eosin (H&E; *A*); TRPA1 antibody (Ab) only (green, arrows point to endothelium; *B*); TRPA1 Ab (green), isolectin Ab (red, endothelium), and 4′,6-diamidino-2-phenylindole (DAPI) (blue, nucleus; *C*) or TRPA1 Ab, TRPA1 blocking peptide, isolectin Ab, and DAPI (*D*). L, lumen. All images were taken at ×200 magnification (scale bar = 100 μm). Summarized data α-pinene (*E*)- and calcitonin gene-related peptide (CGRP; *F*)-stimulated relaxations of PE-precontracted SMA of WT mice without [control; dimethyl sulfoxide (DMSO)] and with CGRP receptor antagonist, SB-268262 (1 µM) (*n* = 3,5 mice, respectively). Values are means ± SE; *n*, 3–5 mice per group. For comparison of two concentration-dependent response curves, a two-way ANOVA with repeated measures and Bonferroni all pairwise post hoc test was used. *P < 0.05; treatment vs. control.

To assess TRPA1 localization, thin sections of SMA were stained for TRPA1 by immunofluorescence (Fig. 8, *A*–*D*). We observed specific TRPA1 immunofluorescent staining as brightest in the endothelium layer (Fig. 8*B*; green), and it was colocalized with the endothelium marker, isolectin (Fig. 8*C*; red). The green staining was absent in the presence of a TRPA1 blocking peptide indicating antibody selectivity (Fig. 8*D*). Because the α-pinene-induced relaxation was dependent on TRPA1 and there was positive TRPA1 staining both in the endothelium and sparsely in the vascular wall (Fig. 8*B*), we tested whether TRPA1 activation was dependent on vasodilator CGRP action. Addition of a CGRP receptor antagonist (SB-268262, 1 µM) did not block the α-pinene-induced relaxation (Fig. 8*E*), however, it did significantly inhibit the CGRP-induced relaxation in PE-precontracted SMA as a positive control (Fig. 8*F*). This result showed that α-pinene-induced relaxation in SMA was independent of CGRP.

## DISCUSSION

To our knowledge, this is the first study to show the vasoreactivity of the α-pinene-derived alcohol metabolites, myrtenol and verbenol, and to detail their mechanisms of action in isolated blood vessels. Our results show that both myrtenol and verbenol stimulate equally potent relaxation in SMA as that of parent α-pinene indicating that the robust relaxation of α-pinene may be a consequence of its metabolism. This is a novel finding because, as far as we know, the effects of inhaled bVOCs have not been previously attributed to their metabolites. Hence, this finding underscores the value of studying the physiological actions of bVOCs that are abundant in the natural (forests) and urban environments (parks, street trees, residential greenness). In addition, bVOCs are also present in high abundance in foods, beverages, and numerous consumer products, such as cosmetics, air fresheners, etc., and thus, the human exposure to bVOCs (and consequently their metabolites) is likely to be frequent and robust.

We found that inhalation exposure of mice to 1 ppm α-pinene increases its alcohol metabolites, myrtenol (primary alcohol) and verbenol (secondary alcohol), indicating that inhaled (and likely ingested) α-pinene can be a prominent source of these metabolites. Moreover, we show that these metabolites are not produced endogenously in mice under laboratory housing conditions with corn cob-derived bedding, whereas the use of pine-sourced bedding may be different. Notably, the 1 ppm level we used is not exceedingly high because levels of personal exposures to multiple monoterpenes including α-pinene, β-pinene, and Δ^3^-carene are documented from 1.8 to 38 ppm in Swedish joinery shops, for example (27). Our estimates of circulating blood levels of α-pinene metabolites suggest that these are likely to be in the low end of the vasoactive range, a finding that aligns well with the observed sensitivity of the SMA to environmental aldehydes, such as acrolein, crotonaldehyde and cinnamaldehyde, as well (26, 28).

Our results are consistent with the notion that at low circulating levels α-pinene and its metabolites activate the endothelium and stimulate formation and release of NO. This increase in NO induces relaxation in VSMC via activation of soluble guanylyl cyclase (see Graphical abstract). In addition to NO’s regulation of blood pressure and blood flow via diminished vascular resistance, NO inhibits vascular inflammation, platelet activation, atherosclerosis development, and plaque rupture, all of which are well-documented beneficial effects of NO, which are likely invoked upon inhalation (and/or ingestion) of bVOCs. Because exposure to greenness is associated with decreases in the incidence of myocardial infarction and stroke in humans (1), it is likely that NO liberation by α-pinene and/or its metabolites (and other bVOCs as well) could be one potential mechanism promoting cardiovascular health. Such beneficial effects of vegetation and greenness are being pursued in studies of greenness and cardiovascular outcomes (1, 7, 29, 30).

Importantly, parent α-pinene triggers a robust vasorelaxation (>60% tension reduction from 0.1–300 µM) that is equally potent and robust as its two oxidized metabolites: myrtenol and verbenol. Moreover, both metabolites stimulate a largely shared mechanism of action with parent α-pinene that is dependent on: intact endothelium, eNOS/NO, and sGC/cGMP. We show that endothelial impairment by air perfusion, by l-NAME addition (chemical inhibition of eNOS), or by eNOS deletion (eNOS-null mice) significantly inhibits the relaxations of α-pinene, myrtenol, and verbenol (see Figs. 3–5). Thus, as indicated above, perhaps the majority of the relaxation of α-pinene may be attributable to its metabolites, a novel consideration. Metabolism of parent pinene (likely in the liver; e.g., cytochrome-P450) generates both the primary (myrtenol) and secondary (verbenol) alcohols, and although we did not focus on where in the body metabolism occurs, a role of vascular wall metabolism is plausible as we observed previously with ethanol- and acetaldehyde-induced relaxations in murine SMA (31). Collectively, these findings support the idea that α-pinene, an abundant and common inhaled and ingested bVOC, and its Phase I metabolites can activate a sensitive, endothelium-dependent vasorelaxation in SMA, yet not in the aorta.

Although 1 mM α-pinene did robustly relax both SMA and aorta (>90%), it led to vasotoxicity in both blood vessels (see Table 1). These data reinforce that the high sensitivity of SMA to α-pinene (and its metabolites) is likely relevant to its physiological function. Specifically, the SMA dilates and increases blood flow to the gastrointestinal tract during/after feeding (postprandial hyperemia)(31), and thus, teleologically, this evolutionarily conserved pathway may augment the digestion of plant material, which contains bVOCs. This may explain why the SMA is six times more sensitive than the aorta. Regardless, evaluating the sensitivity of and the mechanism of action of α-pinene and its metabolites in the SMA is a first step toward a better understanding of how environmental exposures to α-pinene (and perhaps other bVOCs) may influence cardiovascular physiology and health.

This endothelial-dependent relaxation pathway stimulated by α-pinene is shared with several environmental aldehydes such as acrolein, crotonaldehyde, and cinnamaldehyde. As these aldehydes are agonists of TRPA1, (wherein opening TRPA1 increases Ca^++^ permeability, and Ca^++^ activates eNOS; Graphical abstract), we asked whether α-pinene similarly activated TRPA1 in SMA. Because TRPA1 is activated by inhaled irritants (32), it is reasonable to hypothesize that bVOCs may activate TRPA1 (14, 33, 34). Moreover, as some blood vessels express functional TRPA1 in the endothelium, we tested for this possibility in SMA (28, 35). Our results suggest that the mechanism of action of α-pinene (but not its metabolites) is, in part, TRPA1-dependent in SMA, and likely TRPA1 present in the endothelium (see Fig. 8; Graphical abstract). It is possible that TRPA1 is also present in other parts of the vascular wall (e.g., perivascular sensory nerves; see positive TRPA1 staining; Fig. 8*B*), and activation of sensory TRPA1 prompts release of vasodilatory peptide, CGRP (36). In our current study, we show that the CGRP receptor antagonist, SB-268262, did not inhibit α-pinene- yet did inhibit CGRP-induced relaxation in SMA (as expected; positive control) (see Fig. 8, *E* and *F*). These data strongly support a necessary role of TRPA1 in the endothelium rather than in sensory nerves in the α-pinene-induced relaxation of SMA.

Although unsaturated aldehydes activate TRPA1 via covalent modification of intracellular amino-terminal free cysteines (37), it is unclear how α-pinene activates TRPA1. Nonetheless, α-pinene stimulates a TRPA1-dependent response in a sensitive concentration range (<30 µM) that is likely relevant to low, circulating, bioactive levels of inhaled (and ingested) α-pinene, although actual blood levels of metabolites need to be quantified in future studies. In comparison, known agonists of TRPA1, including acrolein, AITC, cinnamaldehyde, and crotonaldehyde, activate TRPA1-dependent relaxation in murine SMA in the low µM range, and those relaxations are inhibited either by A-967079 or by TRPA1 deletion (26, 28). In contrast to those findings, we find herein that while TRPA1 deletion significantly prevents relaxations of α-pinene (4×), the TRPA1 antagonist, A-967079, has no effect (see Table 3). Because α-pinene differs chemically from unsaturated aldehydes, we expect that α-pinene activates TRPA1 by a different mechanism that is not blocked by A-967079, which stabilizes the closed conformation of the cation pore akin to a plug as proposed (38, 39). Our previous use of A-967079 (1 µM) in isolated murine SMA showed it similarly and significantly antagonizes relaxations of acrolein (3×), AITC (4×), cinnamaldehyde (3×), and crotonaldehyde (3×) (28). Notably, though, the SMA of TRPA1-null mice is quite insensitive to relaxations of acrolein (8×), AITC (4.5×), cinnamaldehyde (30×), and crotonaldehyde (4×) compared with SMA of WT mice (with and without A-967079) (28) suggesting that TRPA1-null mice provide more definitive evidence of TRPA1-dependence than does use of A-967079 (at 1 µM) alone. Regardless, and despite this mechanistic ambiguity, the sensitivity and efficacy of verbenol and myrtenol in SMA are equivalent to that of parent α-pinene, and this may be a potential therapeutic pathway worth exploring in future studies.

Overall, there are minimally two distinct pathways that contribute to the full vasorelaxation of α-pinene, myrtenol, and verbenol in SMA: One pathway is a sensitive and likely physiological endothelial-dependent pathway (Graphical abstract); and, a second pathway is an insensitive and toxic pathway that likely targets VSMC (Table 1). We did not investigate the mechanism underlying second pathway because it is a toxic pathway that occurs at levels of α-pinene (1 mM) unlikely to be achieved in vivo, and thus, it is irrelevant to the physiological effects of bVOCs, the focus of this study.

In conclusion, this study describes a sensitive α-pinene-induced vasorelaxation of SMA that is sequentially dependent on the endothelium, TRPA1 channel, NO release, and sGC. This pathway may play an important role in regulating blood pressure/blood flow of inhaled (and ingested) bioactive α-pinene (and perhaps other bVOCs), although this remains to be shown. Furthermore, this pathway (except TRPA1) is activated by α-pinene metabolites, myrtenol and verbenol, a novel finding. Future studies of the quantitative contribution of inhaled α-pinene (and other bVOCs) and their metabolites to cardiovascular physiology in vivo are required to better understand how inhaled bVOCs (as a correlate of exposure to greenness) positively influence cardiovascular health (40).

## DATA AVAILABILITY

Data will be made available upon reasonable request.

## GRANTS

This research was funded by National Institutes of Health Grants GM127607 (to A.B.), ES033323 (to D.J.C.), OD026840 (to P.L.), ES029846 (to A.B.), and ES030283 (to D.J.C.) and the Jewish Heritage Fund for Excellence (to D.J.C.).

## DISCLOSURES

No conflicts of interest, financial or otherwise, are declared by the authors.

## AUTHOR CONTRIBUTIONS

L.J., Z.X., A.B., and D.J.C. conceived and designed research; L.J., Z.X., P.L., A.B., and D.J.C. performed experiments; L.J., Z.X., P.L., A.B., and D.J.C. analyzed data; L.J., Z.X., P.L., S.S., A.B., and D.J.C. interpreted results of experiments; L.J., Z.X., P.L., A.B., and D.J.C. prepared figures; L.J., Z.X., and D.J.C. drafted manuscript; L.J., Z.X., P.L., S.S., A.B., and D.J.C. edited and revised manuscript; L.J., Z.X., P.L., S.S., A.B., and D.J.C. approved final version of manuscript.
